# Within and between Population Variation in Epidermal Club Cell Investment in a Freshwater Prey Fish: A Cautionary Tale for Evolutionary Ecologists

**DOI:** 10.1371/journal.pone.0056689

**Published:** 2013-03-04

**Authors:** Aditya K. Manek, Maud C. O. Ferrari, Robyn J. Pollock, Daniel Vicente, Lynn P. Weber, Douglas P. Chivers

**Affiliations:** 1 Department of Biology, University of Saskatchewan, Saskatoon, Saskatchewan, Canada; 2 Department of Biomedical Sciences, WCVM University of Saskatchewan, Saskatoon, Saskatchewan, Canada; Swansea University, United Kingdom

## Abstract

Many prey fishes possess large club cells in their epidermis. The role of these cells has garnered considerable attention from evolutionary ecologists. These cells likely form part of the innate immune system of fishes, however, they also have an alarm function, releasing chemical cues that serve to warn nearby conspecifics of danger. Experiments aimed at understanding the selection pressures leading to the evolution of these cells have been hampered by a surprisingly large intraspecific variation in epidermal club cell (ECC) investment. The goal of our current work was to explore the magnitude and nature of this variation in ECC investment. In a field survey, we documented large differences in ECC investment both within and between several populations of minnows. We then tested whether we could experimentally reduce variation in mean ECC number by raising fish under standard laboratory conditions for 4 weeks. Fish from different populations responded very differently to being held under standard laboratory conditions; some populations showed an increase in ECC investment while others remained unchanged. More importantly, we found some evidence that we could reduce within population variation in ECC investment through time, but could not reduce among-population variation in mean ECC investment. Given the large variation we observed in wild fish and our limited ability to converge mean cell number by holding the fish under standard conditions, we caution that future studies may be hard pressed to find subtle effects of various experimental manipulations; this will make elucidating the selection pressures leading to the evolution of the cells challenging.

## Introduction

Many species of prey fishes, particularly those members of the superorder Ostariophysi, possess large epidermal club cells (hereafter ECCs) in their skin [1], [2]. Understanding the selection pressure leading to the evolution of those cells has been somewhat elusive. Following from the pioneering work of Von Frisch [3], [4], initial experiments focussed on predation-centered hypotheses for the evolution of the cells, but more recently much more emphasis has been placed on immune-centered hypotheses [5].

When the skin of the fish is damaged and the ECCs are ruptured, as would occur during a predator attack, chemicals initiating anti-predator responses in nearby conspecifics are released in the water column. Not surprisingly, these chemicals are often referred to as chemical alarm cues. In a pioneering experiment, Smith [6] established that, during the breeding season, male minnows lose their ECCs and skin extracts made from breeding minnows do not evoke anti-predator behaviour in conspecifics. This finding lead to the conclusion that ECCs are the source of the alarm cues and has been supported by numerous studies [7], [8], [9], [10]. However, a recent study by Carreau-Green et al. [11] suggested that the skin of juveniles of one species of fish may evoke an alarm response in conspecifics even before the cells appear. If this finding is supported by additional experiments, it would provide strong evidence against the role of the cells as the source of alarm cues. Moreover, a recent paper by Mathuru et al. [12] indicates that GAG chondroitin may be a major component of alarm cues in ostariophysan fishes. There is no known link between chondroitins and ECCs, further weakening the conclusion that ECCs may be responsible for evoking the alarm reactions. Alarm cues may also be mixtures of chemicals with different constituents in different parts of the epidermis including the ECCs.

Understanding the evolution of ECCs as production and/or storage areas for alarm cues has been problematic because the sender of the cue needs to be captured in order for the cues to be released. The critical question that needs to be addressed is: what is the benefit to the sender of this signal? Early research has focussed on the potential for kin associations to explain the existence of ECCs [13], [14]. However, there is limited evidence that most fishes shoal with kin or that kin selection could explain ECC evolution [5].

Other predation-centered hypotheses for the evolution of alarm cues suggest that the chemicals may have evolved as predator attractants [15]. Secondary predators attracted to the location of damaged prey may fight over the prey, giving a chance to the captured prey to escape [16]. There is some evidence for the secondary predator attraction hypothesis, but the frequency with which predators would interfere with each other may be rare and hence this explanation is somewhat unsatisfying.

Chivers et al. [17] provided an alternative to the predation-centered hypotheses for the evolution of alarm cues. They suggested that ECCs may act as a first line of defence against pathogens and parasites that penetrate through the skin. Indeed, they showed that exposure to both skin-penetrating pathogens (water moulds) and parasites (larval trematodes) lead to increases in ECC numbers, suggesting that these cells are part of the immune system [17]. Skin infections do not always lead to an increase in ECCs. A study by James et al. [18] showed that minnows exposed to cercariae of a highly specialized minnow trematode *Ornithodiplostomum ptychocheilus*, where able to infect the host without eliciting an increase in ECC investment. Halbgewachs et al. [19] tested the immune system hypothesis by suppressing the immune system of fishes with cortisol and showing that consequently, the number of ECCs dramatically decreased. In a similar experiment, Chivers et al. [17] showed that fish that had their immune systems suppressed with heavy metals (Cd) lost their ability to increase ECC investment upon exposure to pathogens. In this evolutionary scenario, selection to produce ECCs was driven by disease/pathogen dynamics and the anti-predator function of the cells evolved secondarily, because they represent a reliable signal that a conspecific in the vicinity was recently captured by a predator.

A number of experimental studies have identified factors that may be important in determining ECC investment in fishes. For example, Wisenden and Smith [20] showed that fathead minnows fed higher food rations had higher numbers of ECCs than those fed lower rations. Moreover, individuals raised with unfamiliar conspecifics had more ECCs than individuals raised in familiar groups [14]. Environmental stressors including UV radiation, rapid temperature changes and poor water quality have been shown to result in elevated cortisol levels which are indicators of stress levels and are strongly correlated with reduced ECC investment in fish [19], [21], [22]. Epidermal injury induced by handling and transportation can also result in changes in cortisol levels and hence could likewise influence ECC numbers [23].

Despite the wealth of studies showing that specific factors influence ECC investment, there are some notable inconsistencies with researchers being able to document changes in ECC investment. For example, Pollock [24] found inconsistent ECC responses of minnows to pathogenic water moulds. This finding weakens the immune-function hypothesis. It is clear that we still have much to learn about what drives ECC density in fishes. One finding that is immediately apparent from Pollock's work is that the baseline level of ECCs in control treatments was extremely variable. Fish were collected from different populations and were held in the laboratory for different periods of time prior to experimentation. Each of these factors could have led to variation in the baseline level of cells and hence could have compromised her ability to provide strong experimental tests of factors influencing ECC investment. In another study, Michalak [25] completed two different pathogen experiments using minnows caught from the same shoal. Again, she found a large discrepancy in the baseline number of ECCs in the two experiments despite the fact that her control conditions were identical. An obvious source of variation could have been differences in the time the minnows were held in the laboratory.

If we are to develop a comprehensive understanding of factors that drive ECC investment and explain the evolution of these cells, we need to step back and begin to understand the source and magnitude of variation in ECC numbers. Hence the goal of our current work was threefold. First, we tested for differences in ECC investment among four local populations of wild-caught fathead minnows collected at the same time of year. Between-population differences in ECC numbers has been documented by Hugie [26]. Unfortunately, his data presentation does not allow us to understand the magnitude of the differences he observed. The second goal of our study was to test for within-population differences in ECC investment among four sites within a single waterbody. The final goal of our work was to understand if raising fish under standard laboratory conditions could reduce differences in ECC investment and hence be used as a technique for researchers that want to conduct manipulative experiments to test factors influencing ECC investment. This technique could not only be used to reduce differences in ECC number for fish caught from a single population, but could also reduce between population differences and may be a valuable technique to allow tests of how populations with different predation or pathogen exposure respond to experimental manipulations.

## Materials and Methods

### Ethics Statement

This study was carried out in strict accordance with the recommendations of the approved University of Saskatchewan animal care protocol number 20050067. A fish collection permit was obtained from Ron Hlasny, Senior Aquatic Ecologist, Fish & Wildlife Branch of Saskatchewan Environment.

### Fish collection for field survey

Non-breeding adult fathead minnows were collected from four different populations in and near Saskatoon, Saskatchewan, Canada in late fall 2009 using seine nets and minnow traps. We caught the fish outside the breeding season because male minnows in reproductive condition have reduced numbers of ECCs [6]. Feedlot Pond is a 1-ha pond located on the University of Saskatchewan campus in Saskatoon. The pond was originally filled from the South Saskatchewan River in 1959 to provide water for agricultural purposes. Historically, water (and potentially fish) from the river were pumped into the pond on an annual basis, but no water has been pumped into the pond for at least 15 years and consequently it can be considered a closed system. Pike Lake is an oxbow lake of the South Saskatchewan River, located approximately 33 km south of Saskatoon. Water (and possibly fish) are pumped from the river occasionally to maintain water levels in the lake. Both Marshy Creek and Oscar Creek drain into Redberry Lake, a large saline lake within an enclosed evaporation basin approximately 73 km northwest of Saskatoon. Both Marshy Creek and Oscar Creek contain numerous beaver dams and culverts that impede the movement of fish in the creek.

For the field survey, we collected 50 minnows from each of Feedlot Pond, Pike Lake and Oscar Creek populations. In Marshy Creek we collected 50 minnows from each of four locations (hereafter referred as M-1, M-2, M-3 and M-4) separated by approximately 6 to 12 km. Table 1 provides a summary of water quality parameters (water temperature, dissolved oxygen, pH, salinity and conductivity) that were recorded at each site at the time the fish were captured. Immediately after capture, the fish were euthanized with an overdose of MS-222 in accordance with the Animal Care Protocol Number 20050067. The fish were weighed and measured (standard length), and subsequently fixed in 10% neutral buffered formalin (3.7% formaldehyde w/v) until processed for histological analysis. Table 2 provides a summary of the body condition parameters as well as the mean number of blackspots on each fish. Blackspot disease is commonly observed in freshwater fish as pinhead sized spots located on the fins and body of infected fish [21], [27]. It is caused by a trematode parasite (*Ornithodiplostomum sp*.) having a three-host life cycle, where the fish is the second intermediate host [28]. Exposure to trematodes is known to influence ECC investment [17].

**Table 1:** Mean (±S.E.) water quality parameters for field survey.

Group	Temperature(°C)	Dissolved oxygen (mg/L)	pH	Salinity (in ppt)	Conductivity (µS)/cm
Pike Lake	16.7	9.97	7.8	0.2	929
Oscar Creek	11.2	7.2	7.7	0.6	903
Feedlot Pond	15.4	10.7	8.9	0.3	N.A
Marshy Creek -1 (M-1)	8.8	9.68	7.5	0.6	764
Marshy Creek-2 (M-2)	9.5	6.6	7.8	0.7	943
Marshy Creek-3 (M-3)	8.4	7.3	7.5	0.5	668
Marshy Creek-4 (MS-4)	7.3	6.38	7.3	0.5	700

**Table 2:** Mean (±S.E.) body condition index parameters and blackspot burden for fish used in the field survey and lab study.

Group	Days in the Lab	Mass M (g)	Standard length L (cm)	Body Condition Index: M/L^3^*100	Epidermal thickness (μm)	Blackspot burden
Pike Lake	Day 0	1.65±0.11	4.48±0.12	1.9±0.01	26.23±1.76	0.71±0.23
	Day 14	1.65±0.08	4.78±0.07	2.0±0.01	38.40±1.28	0.87±0.21
	Day 28	1.88±0.07	4.64±0.06	2.0±0.01	45.18±1.45	0.51±0.19
Oscar Creek		1.84±0.10	4.56±0.11	1.8±0.01	59.79±1.94	1.73±0.19
Feedlot Pond		1.40±0.11	4.79±0.12	1.2±0.01	56.25±2.81	0.00±0.00
Marshy Creek-1	Day 0	1.49±0.08	4.20±0.09	2.0±0.01	62.35±5.61	1.50±0.17
	Day 14	1.71±0.08	4.62±0.08	1.8±0.01	45.36±3.35	1.28±0.19
	Day 28	1.48±0.12	4.56±0.12	1.6±0.01	37.52±3.00	1.11±0.29
Marshy Creek-2		1.15±0.12	3.71±0.13	2.0±0.02	58.47±2.32	0.30±0.23
Marshy Creek-3	Day 0	0.84±0.08	3.45±0.09	2.0±0.01	60.11±1.73	0.66±0.16
	Day 14	0.83±0.08	3.55±0.09	1.8±0.01	35.72±1.56	1.50±0.23
	Day 28	1.22±0.13	4.01±0.16	1.8±0.01	36.20±2.57	1.00±0.37
Marshy Creek-4		1.74±0.09	4.64±0.10	1.8±0.01	72.59±1.85	0.89±0.18

### Laboratory maintenance study

Minnows from the Pike Lake site and two Marshy Creek sites (M-1 and M-3) were randomly selected for the laboratory study. We collected adult minnows from each of the three sites and transported them to the laboratory. The containers housing minnows were aerated until they were brought to the lab and gradually transferred to tanks ensuring a ±1°C variation in temperature between their containers and aquaria water. Minnows were divided in groups of 10 and placed in 74-L aquaria (60×30×40 cm) each of which was equipped with an airstone. We had a total of 10 tanks of 10 fish from each of the three locations. For statistical purposes, we considered the tank, not the individual fish, as our replicative unit. The minnows were reared under standard laboratory conditions for up to 28 days. They were maintained on a 14:10 hr light:dark cycle and fed Nutrafin^©^ tropical flake food *ad libitum* (guaranteed 46% minimum crude protein, 5% minimum crude fat, 2% maximum crude fibre, 8% maximum moisture) twice daily. We conducted a 10% water change each week and measured water quality parameters every other day to check for temperature, pH, nitrate, nitrite, hardness and chlorine levels.

On day 14, minnows from five randomly chosen aquaria from each population were euthanized with an overdose of MS-222 in accordance with the Animal Care Protocol Number 20050067. After being weighed and measured, these minnows were fixed in 10% neutral buffered formalin until they were processed for histological analysis. Minnows from the remaining five aquaria from each population were euthanized on day 28 for histological analysis.

### Histological preparation

Tissue preparation for the analysis of the minnow epidermis followed the methods described by Hugie [26] with specific modifications [22]. Epidermal samples were taken from the dorso-lateral surface just behind the operculum to the dorsal fin and placed between two biopsy pads in histocassettes and stored in formalin. An automatic tissue processor (MUP1, Modular Vacuum Processor) was used to process the fixed skin tissue in a series of ethanol grades and perfused with paraffin wax. Tissues were then manually embedded in paraffin wax. The resulting tissue, embedded in a paraffin block was sectioned using a rotary microtome (HM330, Heidelberg) at 5μm thickness. Following sectioning, 3–5 sections were placed on a pre-cleaned suprafrost slide (VWR micro slides). After the slides were dried on a slide warmer, they were deparaffinised, rehydrated and then stained with periodic acid Schiff's reagent with Harris' haematoxylin (PAS-H) to darken the mucous cells and the basement membrane (PAS) and the nucleus (haematoxylin), rendering ECCs colourless and easily recognizable [26]. Images of each epidermal cross section were captured with a Zeiss Axioplan Fluorescence Microscope with an AxioCamICc1 (Color, 1.4MP) digital camera at 10× magnification. For each slide, we recorded the following parameters: mean number of ECCs per mm of epidermis, mean epidermal thickness and mean ECC density (number of ECC's per area of epidermis in mm^2^ taking epidermal thickness into account) and using Image J 1.32, an image processing and analysis program (available on the National Institute of Health's web page http://rsb.info.nih.gov/ij/). The observer was blind with respect to the treatment.

### Statistical analysis

Previous studies looking at ECC parameters in fish have used different methods to report ECC parameters. Often researchers provide data on the average number of cells in a given length of epidermis tissue (most often ECCs/mm of skin, [4], [17]). However, given that the epidermis of fishes can vary considerably in thickness, other researchers have instead reported the density of ECCs in the skin (or used epidermal thickness as a co-variable) [10], [18]. Indeed, two fish with the same number of ECCs per mm length of skin could have very different ECC densities. We wanted to compare these approaches by asking whether we would reach the same conclusions using both measures. Consequently, to determine whether the mean number of ECCs per mm or the mean density of ECCs differed among the four populations in our first experiment we used a one-way ANCOVA with blackspot load and body condition as covariables. Body condition was calculated as the Studentized residuals of the regression between ln(length) and ln(mass) of the fish. The values were logged to linearize the relationship between length and weight. For the within population test, we likewise compared the mean number of ECCs per mm and the mean density of ECCs among the four Marshy Creek collection locations with an ANCOVA.

For the laboratory experiment, we were interested in knowing whether holding fish under standard laboratory conditions for an extended period of time would reduce the within- and between-population difference in ECC parameters. We did not know how fast the populations could converge; consequently we collected data at both 14 and 28 days. However, visual inspection of the data made it obvious that the populations did not converge by day 14. Consequently, to keep the analysis simple, we restricted our analysis to only comparisons between days 1 and 28. For day 1 we compared whether the three test groups differed in either mean number of ECCs per mm or the mean density of ECCs. We repeated the same analysis on day 28. We also used a Levene's test to compare whether the coefficient of variation for the populations changed through time.

## Results

An examination of the skin sections revealed striking differences among populations in the mean thickness of the epidermis. Pike Lake fish had a much thinner epidermal layer than those from the other populations (Table 2). Given that the width of the epidermis did not overlap between populations we could not include it as a covariable in our analysis.

**Figure 1:**
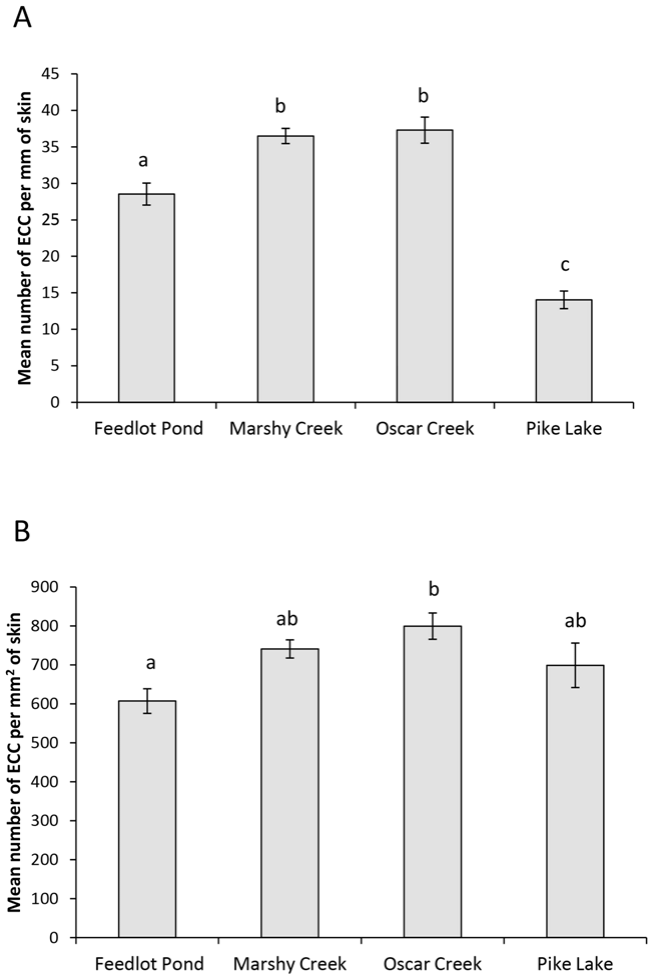
Mean difference in ECC parameters between populations for field survey . Mean (± SE) number of EEC per mm of fish skin (A) and density of ECCs (B) for fathead minnows collected from each of the four populations. Different letters indicate significant differences at P<0.05.

Our results for ECC investment varied depending on whether the data were analysed as ECC/mm or as ECC density. We found an overall significant difference in mean number of ECCs per mm of skin among fish from each of the four populations (ANCOVA: F_3,250_ = 36.6, P<0.001, figure 1A), but neither body condition (F_1,250_ = 0.29, P = 0.59) nor parasite burden (F_1,250_ = 1.4, P = 0.23) accounted for a significant amount of the variance. Tukey post-hoc tests reveal that all populations differed from each other with the exception of Marshy Creek and Oscar Creek. There were nearly three times as many ECCs per mm of epidermis in the Oscar Creek population as compared to the Pike Lake population. Our conclusions about differences among populations in ECCs are very different if we consider ECC density as opposed to number of ECCs per mm. Again, there was an overall difference among populations (ANCOVA: F_3,249_ = 5.26, P = 0.002. figure 1B). Neither body condition (F_1,249_ = 0.29, P = 0.59) nor blackspot burden (F_1,249_ = 1.43, P = 0.23) accounted for a significant amount of the variance. The Pike Lake population did not differ from any of the others with regards to ECC density. Indeed, there were no differences in any of the post-hoc comparisons, except that the Feedlot population had a lower density of ECCs than Oscar Creek and tended to have fewer ECCs than Marshy Creek (p = 0.054).

**Figure 2:**
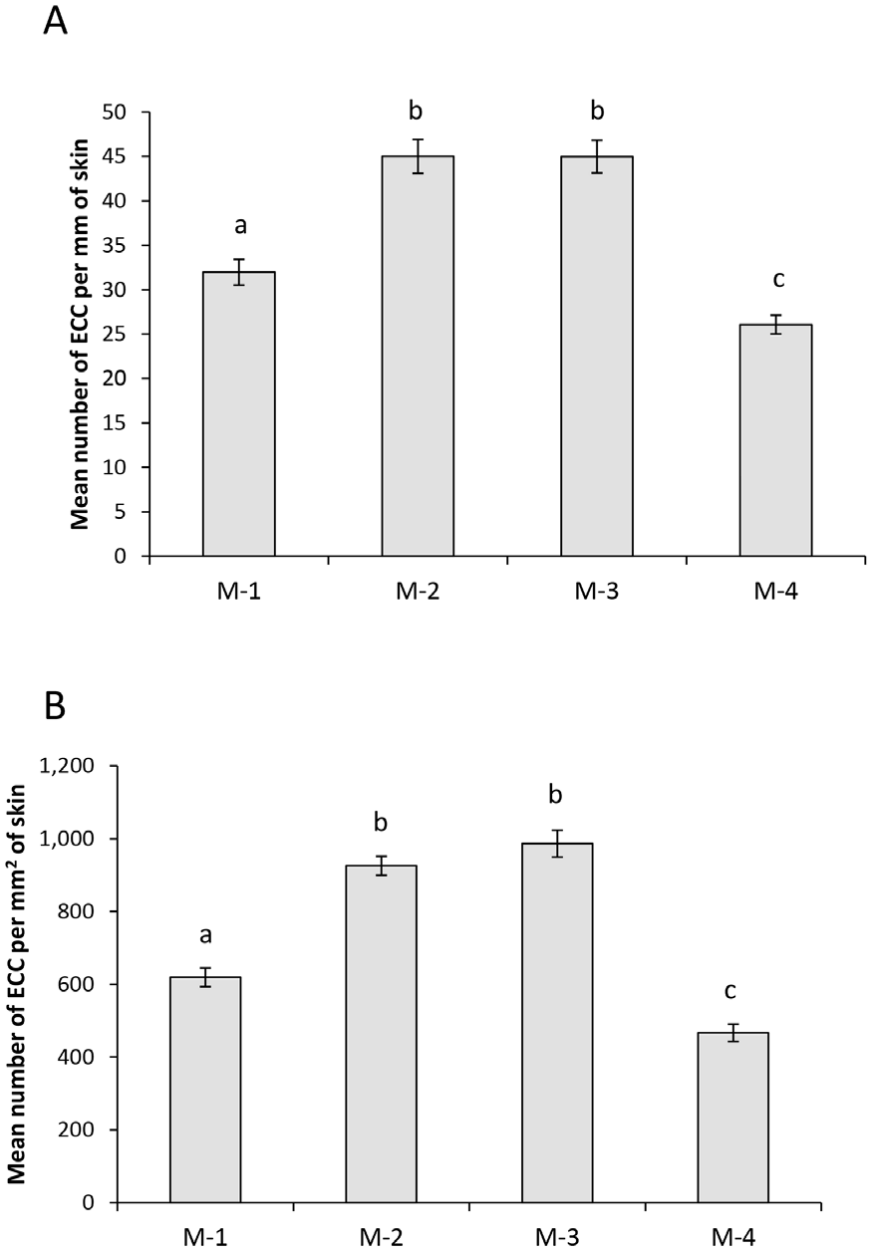
Mean difference in ECC parameters within Marshy Creek locations for field survey . Mean (± SE) number of EEC per mm of fish skin (A) and density of ECCs (B) for fathead minnows collected from each of the four Marshy Creek locations. Different letters indicate significant differences at P<0.05.

Our within-population comparison revealed an overall significant difference in ECCs per mm of epidermis among the four sample locations within Marshy Creek (ANCOVA: F_3,146_ = 32.0, P<0.001, figure 2A) but neither body condition (F_1,146_ = 0.29, P = 0.59) nor parasite burden (F_1,146_ = 0.01, P = 0.93) accounted for a significant amount of the variance. All sample locations differed from each other with the exception of M-2 and M-3 (Tukey tests: P>0.9). Unlike between populations, there is very little difference in epidermal thickness within the Marshy Creek population. This meant that the results we found for difference in ECC density among the 4 sample locations in Marshy Creek matched those looking at number of ECCS per mm of epidermis. Again there was an overall significant difference between sites (ANCOVA: F_3,146_ = 66.9, P<0.001, figure 2B), but neither body condition (F_1,146_ = 0.67, P = 0.42) nor parasite burden (F_1,146_ = 3.6, P = 0.06) accounted for a significant amount of the variance. Each of the sites had an ECC density different than the others except site M-2 and M-3 (P>0.9).

**Figure 3:**
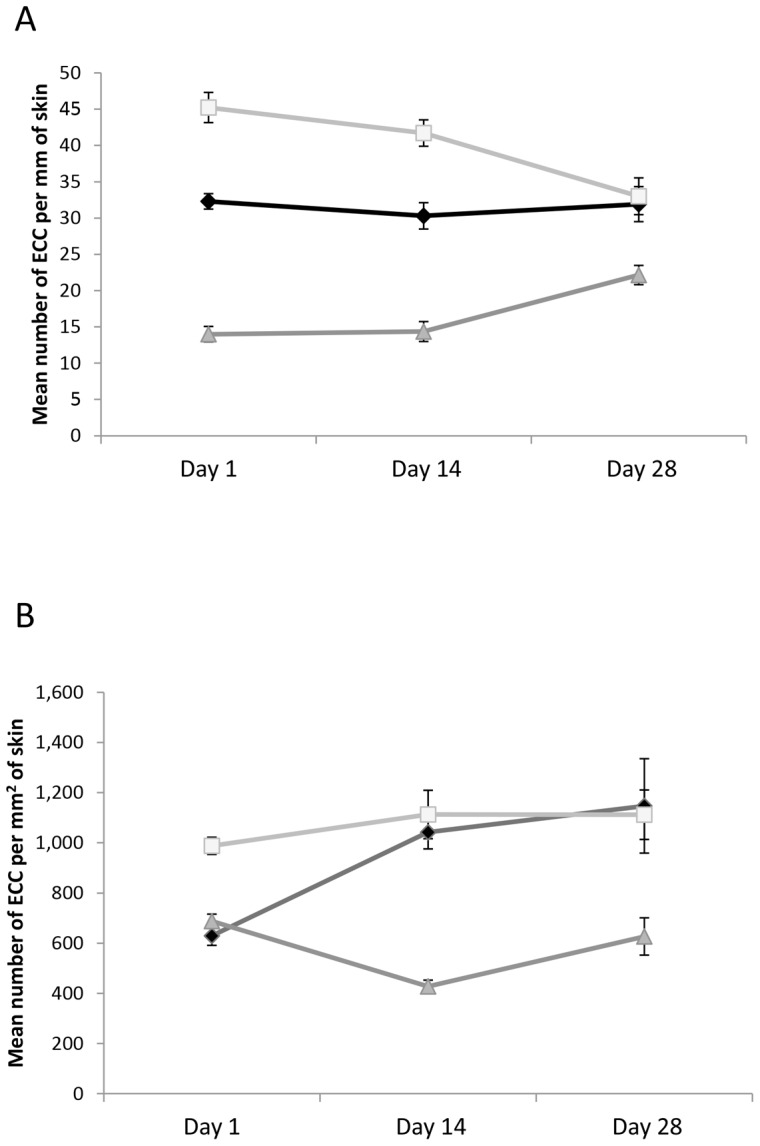
Mean difference in ECC parameters for Laboratory maintenance study . Mean (± SE) number of EEC per mm of fish skin (A) and density of ECCs (B) for fathead minnows collected from Pike Lake (dark gray triangles) and Marshy Creek-1 (black diamonds) and Marshy Creek-3 (grey squares). The graph shows number of ECCs at three points in time (days 1, 14 and 28) after the initiation of the laboratory experiment.

In our laboratory test we found that all three groups [M-1, M-3 and Pike Lake (PL)] of minnows differed in number of ECCs per mm of epidermis at the beginning of the experiment (ANCOVA, F_2,117_ = 65.9, P<0.001, all post hoc tests P<0.001, figure 3A). There was no effect of body condition (F_1,117_ = 0.03, P = 0.87) or parasite burden (F_1,117_ = 0.60, P = 0.44). In contrast, when we consider ECC density, there was an overall difference between the three groups (F_2,12_ = 32.7, P<0.001, figure 3B). Again, body condition (F_1,117_ = 0.54, P = 0.46) and parasite burden (F_1,117_ = 0.24, P = 0.63) did not explain any of the variation. M-1 and PL were both different from M-3 (P<0.001), but similar to each other (P = 0.46). If we consider number of ECCs per mm of epidermis, there was an overall reduction in the difference among populations through time (Levene's test: F_1,193_ = 18.9, P<0.001, Total Var_Day 1_  = 185; Total Var_Day 28_  = 46). By the end of the experiment, we found that there were still significant differences among the three groups (F_2,9_ = 8.4, P = 0.009). The two Marshy Creek populations converged to a similar number of ECCs, but the PL group still had fewer ECCs (Tukey tests: P = 0.009). If we consider ECC density, we find an overall difference at the end of the study (F_2,9_ = 8.6, P = 0.008). Again both Marshy Creek sample locations showed similar ECC densities, but the Pike Lake population differed from both of the Marshy Creek sites (P = 0.011). It is very interesting to observe that there is less difference between the three groups at the end of the experiment than at the beginning of the experiment when we compare the number of ECCs per mm of epidermis. Looking at the range on figure 3, we see that three groups appear to be converging towards a similar value. In stark contrast, when we compare the density of cells between the three groups at the beginning and the end of the experiment, we find that the groups appear to be diverging rather than converging (Levene's test: F_1,193_ = 6.06, P = 0.015, Total Var_Day 1_  = 31188; Total Var_Day 28_  = 82714).

## Discussion

The results of our field survey revealed surprising differences in ECC numbers both within and between populations of minnows. Minnows captured from four different populations showed a threefold difference in mean number of ECCs per mm of skin between Oscar Creek and Pike Lake. We need to be clear that our goal here was not to investigate potential factors that could influence ECC investment between populations, but there were obvious physico-chemical differences between the waterbodies that could contribute to the variation we observed. There was a considerable difference in temperature, but this variable is likely related to the specific weather conditions on the day of collection. We also noted a rather large variation in salinity and conductivity. Other factors that we did not quantify are also likely of considerable importance. For example, in wetlands and lakes in Saskatchewan, dissolved organic carbon (DOC) is known to range from 4.1 to156.2 mg/L [29]. DOC reduces penetration of UV radiation, and according to the results of Manek et al. [22], this differential UV exposure should lead to differences in ECC investment. The other notable factor that could contribute to differential ECC investment is differential levels of pathogens and parasites. We observed both within- and between-population differences in levels of blackspot disease. Minnows are known to increase ECC number in response to trematode infection [17], [18], hence this could contribute to the variation we observed. Likewise, different levels of food resources could lead to variation [20].

More surprising than the between population differences in ECC numbers was the considerable within-population differences. We observed approximately twice the number of ECCs in one of the Marshy Creek sites than in one of the other Marshy Creek sites. This seems like a large difference given that different shoals of minnows were collected within a 12 km stretch of the creek. Physico-chemical characteristics within the creek may be less variable than between the creek and the other waterbodies. Likewise, there is likely less variation in resource levels within a given site than between sites; hence we should expect to find fewer differences in ECCs within populations than between them. Fishes are known to shoal with individuals of similar size, body condition and parasite load [30]. This preferential shoaling may contribute to the considerable variation that we saw between shoals. The site-to-site differences at Marshy Creek could also reflect variation in snail habitat (source of trematode infection) or availability of perches used by hunting kingfishers (source of parasite eggs).

The results from our laboratory experiment showed that fish from different populations responded differentially to our standard laboratory conditions. We observed that minnows from Pike Lake showed no change in number of ECCs per mm through time while some of the Marshy Creek fish increased their ECCs more than others. Our ability to use standard laboratory conditions as a tool to collapse the differences in ECC parameters gave somewhat mixed results. We found that we could converge ECC number/mm and density within a population (Marshy Creek) but not between populations (Marshy Creek vs. Pike Lake). Through time we observed a substantial convergence in the mean number of ECC/mm and the ECC density between the two Marshy Creek sites. This likely indicates that fish from different sites within Marshy Creek show considerable variation in ECCs when they are subject to different local conditions but they start to converge to the same number and density of ECCs when raised in a common environment. The convergence in ECC density within Marshy Creek meant that both the Marshy Creek sites actually diverged from the Pike Lake fish. It remains unknown whether more time in the laboratory would lead to convergence, however, most studies acclimate fish for less than 28 days prior to initiating experiments. Taken together, our results indicate that future researchers need to use extreme caution when attempting to conduct experiments to elucidate factors responsible for driving variation in ECC numbers. If the fish were to have converged to a similar mean value with little variation, then we could expect to be able to find subtle effects of various experimental manipulations. Given the large differences we observed between populations, we may only expect to identify factors that have large effect sizes.

Previous studies examining ECC parameters have reported ECC density while others have reported differences in numbers of ECCs per mm [4], [17], [18], [20]. Surprisingly, we found a large discrepancy in the conclusions we would draw based on these measurements. For example, if we consider number of ECCs per mm, all four of the populations in our field survey differed from each other, whereas if we considered density of ECCs, then only Feedlot Pond was different from Oscar Creek. Likewise, if we look the differences between populations through time, there was convergence in mean number of ECC per mm of epidermis while there was divergence in terms of ECC density. Is one of these measures better than the other? This may depend on the research question that is being asked. Perhaps studies done from a predation perspective may want to use one variable while those done from disease perspective may want to report the other. Our work points to the fact that future researchers need to justify their choice of variables. We suggest that epidermal thickness should be of prime importance when justifying whether mean number of ECCs per mm or mean ECC density should be selected. The mean number of ECCs per mm stands strong under conditions where there is no significant difference in epidermal thickness between treatments.

Hundreds of studies have examined the importance of chemical alarm cues in mediating behavioural, morphological and life history defences in prey animals [31], [32]. Our results have important implications for this work. We suggest that researchers may be inadvertently introducing more variation into their experiments than they realize. For example, many studies have reported that alarm cues are collected by making several vertical cuts along the flank of the fish and then flushing the skin with water to collect the cues [33], [34], [35]. In most of these studies, the researchers use a different donor fish for each replicate. This technique could introduce considerable variation in the amount of alarm cues used in the experiment. This is critical given that several species of fishes, like other prey animals, show threat-sensitive responses to variation in alarm cue concentration [36], [37]. Fish exposed to high levels of alarm cues show very strong responses while fish exposed to lower concentrations show weaker responses. Moreover, fish learn the threat level of predators based on alarm cue concentration present during conditioning [36], [38]. Fish exposed to high concentrations of alarm cues paired with unknown predator odour learn the predator as a high risk, while those exposed to low concentrations of alarm cues, learn the predator as a mild threat. Behavioural ecology is ripe with experiments showing that prey animals show sophisticated behavioural responses to slight variation in risk. Our results indicate that the experimental techniques used to induce variation in risk may be substantial. Many other studies use a single solution of homogenized skin to produce alarm cues [39], [40]. This reduces the variation in alarm cue concentration between replicates in the same experiment. However, we caution that making comparisons between studies is still problematic given the variation that we observed. Another source of variation that was not addressed in our study relates to seasonal variation in ECC investment. Breeding male minnows are known to lose their alarm cues during the breeding season [6], but whether other seasonal variation exists remains unknown. Pathogens, parasites and UV radiation, as well as food level, all vary seasonally and hence could drive seasonal differences in ECC number.

Our work has implications not only for those studying behaviour, but also those that examine predator-induced changes in morphology. Such changes are often triggered by exposure to chemical alarm cues; the investment in morphological defences may be directly linked to perceived threat level [41], [42]. We suggest that the large variation in ECC numbers that we observed provides a source of variation that may be under appreciated by researchers studying morphological change. A similar argument can be made for those researchers that study life history changes induced by alarm cues.

Fathead minnows belong to a large superorder of fish, the Ostariophysi, a group that includes the minnows, characins, catfishes, loaches and suckers. Similar alarm cue systems are also known in other groups of fishes, including the salmonids, cichlids poeciliids and percids [32], [43]. Moreover, alarm cues are known in larval amphibians and numerous taxa of invertebrates. Our cautionary note about within and between population variation in alarm cue concentration likely applies to all of these taxa.
